# Protocol development and feasibility of the PEACH in Asia study: A pilot study on PEri‐anesthetic morbidity in CHildren in Asia

**DOI:** 10.1111/pan.15034

**Published:** 2024-11-09

**Authors:** Soichiro Obara, Choon Looi Bong, Zehra Serpil Ustalar Ozgen, Shemila Abbasi, Ekta Rai, Evangeline K. Villa, Andi Ade W. Ramlan, Raihanita Zahra, Christopher Kapuangan, Komang Ayu Ferdiana, Ina Ismiarti Shariffuddin, Vivian Yuen, Elsa Varghese, Josephine S. K. Tan, Norifumi Kuratani, Asian Society of Pediatric Anesthesiologists (ASPA) research special interest group

**Affiliations:** ^1^ Teikyo University Graduate School of Public Health Tokyo Japan; ^2^ Department of Anesthesia Tokyo Metropolitan Ohtsuka Hospital Tokyo Japan; ^3^ Department of Pediatric Anesthesia KK Women's and Children's Hospital Singapore Singapore; ^4^ Department of Anesthesiology and Reanimation, Faculty of Medicine, Acibadem Altunizade Hospital Acibadem Mehmet Ali Aydinlar University Istanbul Turkey; ^5^ Department of Anesthesia Aga Khan University Hospital Karachi Pakistan; ^6^ Department of Anesthesiology Christian Medical College Vellore India; ^7^ Department of Anesthesiology, Philippine General Hospital University of the Philippines College of Medicine Manila Philippines; ^8^ Department of Anesthesia, Dr. Cipto Mangunkusumo Hospital Fakultas Kedokteran Universitas Indonesia Jakarta Indonesia; ^9^ Department of Anesthesia University of Malaya Kuala Lumpur Malaysia; ^10^ Department of Anesthesiology and Perioperative Medicine Hong Kong Children's Hospital Hong Kong China; ^11^ Department of Anesthesiology Kasturba Medical College Manipal India; ^12^ Department of Anesthesia Saitama Children's Medical Center Saitama Japan

**Keywords:** Asia, child, multicenter study, pediatric anesthesia, quality improvement, sample size

## Abstract

**Background:**

Comprehensive data on pediatric anesthesia outcomes, particularly severe critical events (SCEs), are scarce in Asia. This highlights the need for standardized research to assess anesthesia safety and quality in the diverse settings.

**Aims:**

The PEACH in Asia pilot study aimed to test the feasibility of a standardized protocol for investigating SCEs in anesthesia practices across Asia, evaluate the data acquisition processes, and determine the sample size for a main study.

**Methods:**

This multicenter pilot study involved ten institutions across nine Asian countries, including children from birth to 15 years undergoing diagnostic or surgical procedures. Data on SCEs were collected using standardized definitions. The study assessed the feasibility and estimated the sample size needed for the main study.

**Results:**

The pilot study enrolled 330 patients, with a SCE incidence of 12.4% (95% CI: 9.2–16.4%). Respiratory events were observed in 7.0% of cases, cardiovascular instability in 4.9%, and drug errors in 0.6%. Based on the SCE incidence observed in the pilot study, the estimated sample size required for the main study is at least 10 958 patients. The pilot study demonstrated the feasibility of the study protocol but identified several challenges, particularly in resource‐limited settings. These challenges included a significant burden associated with data collection, technical issues with electronic case report forms (e‐CRFs), variability in patient enrollment across institutions (ranging from 4 to 86 patients per site), and incomplete data acquisition (24.8% of height data and 9.7% of disposition data were missing).

**Conclusions:**

The PEACH in Asia pilot study successfully validated a protocol for investigating SCEs in pediatric anesthesia across Asia. Addressing the challenges identified in the pilot study will be crucial for generating robust data to improve pediatric anesthesia safety in the region. Key issues to address include improving data collection methods, resolving e‐CRF technical difficulties, and ensuring consistent institutional support.

## 1 INTRODUCTION

Pediatric anesthesia poses significant challenges, particularly in low‐ and middle‐income countries (LMICs), where the risks of adverse events and mortality are disproportionately higher than in high‐income countries (HICs).^1^, ^2^ While pediatric anesthesia‐related mortality rates in HICs range from 0.01% to 0.05%, these rates can be up to three times higher in LMICs, highlighting a critical disparity in patient outcomes.^1^, ^2^ Adverse events during pediatric anesthesia occur in 2–8% of cases in HICs,^1^, ^2^, ^3^, ^4^, ^5^, ^6^ although these rates vary widely in Asian countries, from 3.3% in Singapore to 8.9% in India.^7^, ^8^, ^9^ Although tracking surgical and anesthesia outcomes is crucial for guiding improvements in perioperative care,^10^ there is a significant lack of comprehensive research on those outcomes in pediatric surgery and anesthesia in Asian countries or regions, particularly regarding severe critical events (SCEs).^11^ On top of that, the lack of standardized definitions and metrics, like those employed in the APRICOT study,^6^ complicates efforts to accurately benchmark and improve safety in this context.^12^

Asia is a particularly challenging region for achieving universal coverage of safe pediatric anesthesia. Nearly 60% of the world's population and more than one billion children live in the region, yet many of these children lack access to specialized anesthesia care.^13^, ^14^ According to the World Federation of Societies of Anesthesiologists (WFSA) anesthesia workforce survey, many Asian countries have a low density of anesthesia providers, further exacerbating the difficulty of providing safe anesthesia to children in these regions.^15^ To deliver safe pediatric anesthesia across the region, it is crucial to establish a comprehensive understanding of the current state of pediatric anesthesia in the region, including the incidence and causes of SCEs.

To address these challenges, the Asian Society of Pediatric Anesthesiologists (ASPA) launched the PEACH (PEri‐Anesthetic morbidity in CHildren) in Asia study project. The primary aim of the PEACH in Asia study is to investigate the incidence and causes of SCEs in children undergoing anesthesia or sedation across multiple Asian countries for guiding improvements in perioperative care as well as education and training of caregivers. The study seeks to standardize definitions and data collection methods to ensure consistency across the diverse settings. Additionally, it aims to describe the diversities in pediatric anesthesia practices throughout Asia and assess the impact of this variability on the incidence of SCEs. As a first step toward achieving this goal, we developed the protocol for the PEACH in Asia study and tested the feasibility of our protocol across diverse Asian countries or regions. We also aimed to provide a reasonable estimate of the sample size needed for the main study.

## 2 METHODS

### 2.1 Protocol Development for the PEACH in Asia Study Project

The PEACH in Asia study is fully supported and endorsed by the ASPA, which has over 700 members across 30 Asian countries. The ASPA established a research special interest group that led the development of the draft protocol for PEACH in Asia study data acquisition. The development process involved a comprehensive review of existing literature, consultations with pediatric anesthesia experts across Asia, and iterative revisions based on preliminary feedback. Being inspired by the APRICOT study conducted in Europe,^6^ the protocol of the PEACH in Asia study was designed to establish a consistent approach to data collection and outcome measurement, enabling accurate benchmarking of pediatric anesthesia safety. Special consideration was given to the diverse range of clinical practices across the region, prioritizing ease of implementation in light of language barriers and resource limitations.

### 2.2 PEACH in Asia study protocol

#### 2.2.1 Study design

This study is a multinational, multicenter, observational study involving children from birth to 15 years old undergoing elective or emergency anesthesia for diagnostic or surgical procedures. It is modeled after the APRICOT study conducted in Europe.^6^ The full study protocol is posted on a preprint server (medRxiv) to enhance transparency, with revision made as necessary.^16^

#### 2.2.2 Study setting

The study welcomes participation from any hospital involved in pediatric anesthesia across Asian countries.

Inclusion Criteria

- Age: Children from birth to 15 years old.
- Procedures: Includes all inpatient or outpatient procedures (either surgical or non‐surgical) requiring general anesthesia or sedation with or without regional analgesia, or regional anesthesia alone.

Exclusion Criteria

- Age: Children aged 16 years or older.
- ICU Patients: Children transferred directly from pediatric intensive care units to the operating room.
- ICU Procedures: Anesthesia or sedation administered in neonatal or pediatric intensive care units.

#### 2.2.3 Clinical outcome measures

The primary endpoint is any SCE requiring immediate intervention, which could lead to major disability or death. SCEs include laryngospasm, bronchospasm, pulmonary aspiration, drug errors, anaphylaxis, cardiovascular instability, perioperative cardiac arrest, neurological damage, and postoperative stridor. The time frame for these events is from the onset of anesthesia to 60 minutes post‐procedure (see Table 1 for definitions, aligned with APRICOT criteria).^6^

**TABLE 1:** Definitions of severe critical events.

1	Bronchospasm	An increased respiratory effort, especially during expiration, and wheeze on auscultation. If the patient is ventilated, bronchospasm may also be considered if a significant increase in peak inspiratory pressure (under volume controlled ventilation) or significant decrease in tidal volume (under pressure controlled ventilation) are observed. In all cases, any episode of airway constriction requiring the administration of a bronchodilator will be recorded.
2	Laryngospasm	Complete airway obstruction associated with rigidity of the abdominal and chest walls and leading to unsuccessful child's ventilation, or glottic closure associated with chest movement but silent unsuccessful child's respiratory efforts and assisted ventilation, unrelieved in both situations with simple jaw thrust and continuous positive airway pressure (CPAP) maneuvers and requiring the administration of medication (propofol, fentanyl, suxamethonium, rocuronium etc.) and/ or tracheal intubation.
3	Pulmonary aspiration	The presence of any non‐respiratory secretions (bilious or particulate) in the airway as evidenced by laryngoscopy, suctioning, or bronchoscopy. In a situation where there was suspicion of pulmonary aspiration but no positive aspiration of non‐respiratory secretions, new clinical and/ or chest X‐ray signs consistent with aspiration are accepted as evidence for it (e.g., new wheeze or crackles in the chest after regurgitation or vomiting incident).
4	Drug error	The administration of a wrong drug, or a wrong dose given by any route, or a wrong site of administration, that has led to either respiratory/ cardiac/ neurological consequence or to an unplanned admission to the intensive care unit(ICU) or prolonged hospitalization.
5	Anaphylaxis	The occurrence of any suspected IgE or non‐IgE mediated severe allergic reaction leading to cardiovascular instability and/or severe bronchospasm and requiring immediate resuscitation (fluid resuscitation and epinephrine).
6	Cardiovascular instability	The occurrence of either one of the following: Cardiac arrhythmia defined as electrocardiogram(ECG) evidence of cardiac rhythm disturbance considered by clinical staff to be severe enough to require treatment (e.g., anti‐arrhythmic agents, vasoactive agents, intravenous fluid, etc.). This includes arrhythmias occurring following regional analgesia and requiring intervention. For example: bradycardia requiring atropine, supraventricular tachycardia, atrial or ventricular tachyarrhythmia, torsade de pointe, etc. Hypotension defined as a drop in blood pressure requiring intervention by the anesthesiologist (fluid resuscitation and/or the administration of vasoactive drugs). Bleeding resulting in hypotension and necessitating unanticipated and unpredicted blood transfusion. Cardiovascular instability despite anticipated bleeding and transfusion (e.g., liver transplant, scoliosis…).
7	Cardiac Arrest	Cessation of circulation (e.g., pulseless electric activity, asystole, ventricular fibrillation/tachycardia) requiring open or closed chest compressions, or resulting in death, while the patient is in the care of the anesthetic team.
8	Neurological damage	In case of regional anesthesia: the occurrence of nerve injury or spinal cord insult or seizure requiring resuscitation. In case of general anesthesia: any episode of seizure, pressures sore, episodes of loss of vision or new onset of central neurological impairment. This includes peripheral nerve injury following positioning (ulnar nerve, external popliteal nerve) or puncture (median or ulnar nerve).
9	Stridor (Post‐operative)	A severe inspiratory flow limitation with sternal retraction, intrathoracic pressure swing, and potentially cyanosis occurring in the post‐anesthesia care unit (PACU) and necessitating the administration of oxygen, intravenous steroids and/or epinephrine (nebulization) or tracheal intubation.

#### 2.2.4 Data collection and management

Paper case report forms (p‐CRFs) is used for standardized data recording (Appendix S1). Each institution receives a country code and a hospital code, and assign a unique patient study ID. Data are entered anonymously into a secure, cloud‐based electronic case report form (e‐CRF) on UMIN‐INDICE, an internationally approved platform. Data confidentiality are maintained according to Good Clinical Practice Guidelines.

Data collection takes place over a period of two consecutive weeks including weekends and after‐hours. The 2‐week recruitment period is chosen by each participating institution during planned study periods of the main study.

#### 2.2.5 Statistical methods

The data analysis involves descriptive statistics to summarize patient demographics and the incidence of SCEs. Exploratory statistical analyses, including hierarchical logistic regression or relative risk models, will be conducted to identify independent risk factors for SCEs, with results reported as adjusted odds ratios or relative risks.

Participating institutions will be allowed to request to use their data for secondary analyses. Proposals for such analyses should be submitted to the ASPA research special interest group, which will review and approve the proposals.

#### 2.2.6 Ethics approval statement

This study adheres to the ethical principles outlined in the Declaration of Helsinki and the International Conference on Harmonization Good Clinical Practice guidelines. Ethical approval has been granted by the Institutional Review Board (IRB) at Tokyo Metropolitan Ohtsuka Hospital, Tokyo, Japan, for this international prospective observational multicenter study. Ethics requirements may vary by country and institution; therefore, all participating sites must obtain formal ethical approval or waivers as appropriate before commencing the study.

#### 2.2.7 Clinical trial registration

The study is registered with the University Hospital Medical Information Network (UMIN) under the identifier #UMIN000046328, registered on December 28, 2021.

#### 2.2.8 Informed consent

As an observational study without interventions, this study does not modify routine clinical care. Participating institutions need to obtain necessary IRB approvals or consent waivers as required by their local regulations. If informed consent is required according to their local regulations, informed consent forms, information for parents/guardians, and recruitment advertisements must be IRB‐reviewed and approved where applicable.

#### 2.2.9 Sample size calculation for the main study

The following formula was used to estimate the required sample size (***n***) for the main study:

$$n=\left\{Z^{2}\cdot p\cdot \left(1-p\right)\right\}/E^{2}$$

where: **Z** is the *Z*‐value corresponding to the desired confidence level (1.96 for 95% confidence, 2.576 for 99% confidence). **p** is the anticipated incidence rate, which is derived from the SCE rate of the pilot study. **E** is the allowable margin of error, which can be calculated as the half of the confidence interval (CI) width when the CI width is known.

Using the APRICOT study's reported SCE rate of 5.2% [95% CI: 5.0, 5.5],^6^ the CI width was approximately 10% (±5%) of the incidence rate. Thus, **E** is set to 5% of the SCE rate, which wderived from the pilot study results.

#### 2.2.10 Roles of research coordinators

National Coordinators: Responsible for:

- Identifying local coordinators at participating institutions
- Assisting with translation of study documents
- Ensuring IRB approvals are in place appropriately
- Facilitating communication between participating institutions

Local Coordinators: At individual institutions, responsible for:

- Leading the study locally
- Ensuring IRB approvals locally
- Training staff for data collection
- Overseeing daily data collection and problem‐solving.

### 2.3 Pilot study

#### 2.3.1 Study objectives

The pilot study was designed to assess the feasibility of the PEACH protocol and to evaluate the data acquisition processes. Its primary objectives were to test these processes in a real‐world setting and to provide a basis for determining an appropriate sample size for the subsequent full‐scale study.

#### 2.3.2 Study design

The pilot study utilized a design closely aligned with that of the main study, incorporating identical inclusion and exclusion criteria, outcome measures, and data collection methodologies, including the use of CRF and the cloud‐based data entry system. However, the pilot study's recruitment period was shortened to 3 days, in contrast to the two‐week period planned for the main study.

#### 2.3.3 Study setting

The pilot study was conducted at ten institutions across nine countries or regions in Asia, including Türkiye, Pakistan, India, Indonesia, Singapore, Malaysia, the Philippines, Hong Kong, and Japan (with two institutions in Japan) between May 1st and June 30th, 2023. Those institutions were selected based on their affiliation with members of the ASPA research special interest group.

#### 2.3.4 Ethics approval statement

Ethical approval for the pilot study was granted by the IRB at Tokyo Metropolitan Ohtsuka Hospital, Tokyo, Japan, as part of the PEACH in Asia study project. All participating institutions also obtained the necessary ethical approvals or waivers as required from each institutional IRB.

#### 2.3.5 Analysis

Descriptive statistics were utilized to summarize patient characteristics and primary outcomes. The pilot study did not involve imputation of missing data. Data on the incidence of SCEs were used to estimate the sample size for the main study.

## 3 RESULTS

### 3.1 Feasibility findings

The pilot study successfully enrolled 330 patients across nine institutions in nine countries or regions. However, one institution in Japan withdrew from the study due to the substantial burden of data collection and entry into the e‐CRF, compounded by the research coordinator's clinical workload during the three‐day recruitment period.

Support materials, including newsletters, infographics, and user guides for e‐CRF entry, were distributed from the principal investigator to the research coordinators. Translation of these materials into local languages was essential for obtaining IRB approvals in Türkiye, Indonesia, the Philippines, and Japan. The local IRB granted a waiver of consent, except for the Philippines. At the participating institution in the Philippines, consent was obtained for 23 cases among 25 eligible cases identified during the three‐day period, yielding a consent rate of 92%.

Significant variation was noted in patient enrollment across institutions: 86 patients from Indonesia, 68 from Singapore, 42 from India, 36 from Türkiye, 32 from Hong Kong, 25 from Malaysia, 23 from the Philippines, 18 from Pakistan, and 4 from Japan. This variation was attributed to the differing types of institutions involved, such as children's hospitals versus general hospitals.

Among the ten coordinators who collected data, two encountered difficulties with data entry into the e‐CRF, while the remaining coordinators successfully entered data into the e‐CRF with using the provided user guide. While the majority of e‐CRFs were completed, there were some data gaps: 24.8% (82 out of 330) of height data, 9.7% (32 out of 330) of disposition data, and 1.8% (6 out of 330) of weight data were missing, with no imputation performed for this analysis.

### 3.2 Clinical outcome measures

Of the total enrolled 330 patients, 290 (87.9%) were of Asian descent, and 64 (19.4%) had an American Society of Anesthesiologists (ASA) physical status of III or higher. Detailed patient demographics, medical history, anesthesia management, and procedures are presented in Tables 2 and 3.

**TABLE 2:** Baseline characteristics of the registered patients.

		Overall		Critical Events (+)		Critical Events (−)	
		*n* = 330		(*n* = 36)		(*n* = 294)	
			SD or *n* (%)		SD or *n* (%)	Total	SD or *n* (%)
		*N*	(%)	*N*	(%)	*N*	(%)
Age of the patient							
Preterm neonatal		7	2.1	3	8.3	4	1.4
From birth ≦27 days (Term neonatal)		6	1.8	2	5.6	4	1.4
≧28 days, ≦12 months (Infant)		53	16.1	6	16.7	47	16.0
≧13 months, < 2 years (Toddler)		20	6.1	3	8.3	17	5.8
≧2 years, < 6 years (Early childhood)		92	27.9	14	38.9	78	26.5
≧6 years, < 12 years (Middle childhood)		101	30.6	4	11.1	97	33.0
≧12 years, < 16 years		51	15.5	4	11.1	47	16.0
Sex							
Male: Female		186: 143	56.4	24: 12	66.7	164: 131	55.8
(Unknown: 1)			(male %)		(male %)	(unknown 1)	(male %)
Ethnicity							
Asian		290	87.9	35	97.2	285	96.9
White		35	10.6	1	2.8	34	11.6
Arabic		2	0.6	0	0.0	2	0.7
Other		3	0.9	0	0.0	3	1.0
ASA‐PS							
1		119	125	37.9	10	27.8	115
2		123	132	40.0	11	30.6	121
3		44	56	17.0	10	27.8	46
4		1	1	0.3	0	0.0	1
1E		4	5	1.5	1	2.8	4
2E		2	4	1.2	1	2.8	3
3E		3	4	1.2	1	2.8	3
4E		2	3	0.9	2	5.6	1
Weight	mean (SD)^a^	21.9	(16.8)	11.8	(7.2)	22.8	(17)
Airway sensitivity							
Upper respiratory tract infection in the past 2 weeks		24	10.4	2	5.6	22	7.5
Wheezing in the past 12 months		14	6.1	7	19.4	7	2.4
Asthma diagnosis		5	2.2	0	0.0	5	1.7
Passive smoking		14	6.1	1	2.8	13	4.4
Airway sensitivity^b^		50	21.7	8	22.2	42	14.3
Environmental sensitivity							
Allergy		11	3.3	3	8.3	8	2.7
Atopy		14	4.2	1	2.8	13	4.4
Environmental sensitivity^c^		24	7.3	4	11.1	20	6.8
Physical condition							
Prematurity		29	8.8	5	13.9	24	8.2
Snoring		14	4.2	0	0.0	14	4.8
Medication		52	15.8	4	11.1	48	16.3
Handicap		45	13.6	6	16.7	39	13.3
History of past anesthetic complication		8	2.4	1	2.8	7	2.4
ASA‐PS III/IV/V		64	19.4	13	36.1	51	17.3
Physical condition^d^		136	41.2	16	44.4	120	40.8
Anesthesia/Sedation time							
Opening hours vs. After hours/weekends/ holidays		316: 14		34: 2		282: 12	
Type of Procedures							
*Surgical vs. non‐surgical*		242: 88	73.3	25: 11	69.4	217: 77	73.8
			(surgical, %)		(surgical, %)		(surgical, %)
Surgical		244	73.3	25	69.4	219	74.5
Neurosurgery		3.9	2	5.6	11	3.7	4.2
Head and Neck		2.7	1	2.8	8	2.7	3.0
Ophthalmology		37	11.2	0	0.0	37	12.6
Ear‐Nose‐Throat		26	7.9	5	13.9	21	7.1
Plastics (including cleft palate & lip)		30	9.1	3	8.3	27	9.2
Cutaneous/Dermatology		5	1.5	1	2.8	4	1.4
Cardiac surgery		6	1.8	2	5.6	4	1.4
Thoracic		1	0.3	0	0.0	1	0.3
Gastro/Abdominal/Hepato‐biliary/Pancreas		45	13.6	8	22.2	37	12.6
Urological/Kidney		36	10.9	2	5.6	34	11.6
Orthopedic		34	10.3	1	2.8	33	11.2
Trauma		2	0.6	0	0.0	2	0.7
Non‐surgical		88	26.7	11	30.6	77	26.2
Ophthalmologic examination		0.9	0	0.0	3	1.0	1.1
Dental		3.9	3	8.3	10	3.4	3.4
Bronchoscopy		0.6	0	0.0	2	0.7	0.8
Gastroenterology		4.5	5	13.9	10	3.4	3.8
Biopsy		0.6	0	0.0	2	0.7	0.4
Bone Marrow & Lumbar puncture		2.1	0	0.0	7	2.4	2.7
CT‐Scan		0.6	0	0.0	2	0.7	0.8
MRI (Magnetic rad. Imaging)		7.0	1	2.8	22	7.5	7.6
Venous access		0.6	0	0.0	2	0.7	0.8
Burns dressing		0.3	0	0.0	1	0.3	0.4
Other non‐surgical		5.5	2	5.6	16	5.4	4.9
Patient type							
Inpatient: Outpatient		226: 104	68.5	26: 10	72.2	200: 94	68.0
			(Inpatient, %)		(Inpatient, %)		(Inpatient, %)
The senior anesthesiologist in charge							
Specialist anesthesiologist with mainly pediatric practice (>50%)		166	50.3	10	27.8	156	53.1
Specialist anesthesiologist with occasional pediatric practice (<50%)		91	27.6	16	44.4	75	25.5
Anesthesiologist in training		73	22.1	10	27.8	63	21.4
Experience	Mean (SD) [years]	14.6	(10.2)	11.8	(7.2)	15	(10.5)

^a^ Missing in six cases, which were not imputed.

^b^ Airway sensitivity: upper respiratory tract infection in the past 2 weeks, wheezing, asthma, or passive smoking.

^c^ Environmental sensitivity: allergy or atopy.

^d^ Physical condition: prematurity, snoring, medication, handicap, history of past anesthtic complication, or ASA status greater than II.

**TABLE 3:** Anesthesia plans and procedures.

	Overall		Critical Events (+)		Critical Events (−)	
	(*n* = 330)		(*n* = 36)		(*n* = 294)	
		SD or *n* (%)		SD or *n* (%)		SD or *n* (%)
	*N*	(%)	*N*	(%)	*N*	(%)
Anesthesia/sedation practice						
Pre‐medication	59	17.9	11	30.6	48	16.3
Midazolam (oral)	18		1		17	
Acetaminophen (oral)	17		1		16	
Dexmedetomidine nasal	4		1		3	
Other	35		8		27	
Parental presence at induction	182	55.2	21	58.3	161	54.8
Monitoring						
Electro‐encephalogram (EEG) derived data (e.g., BIS)	19	5.8	0	0.0	19	6.5
Induction						
Inhalational	180	54.5	16	44.4	164	55.8
Intravenous	149	45.2	19	52.8	130	44.2
Intramuscular	1	0.3	1	2.8	0	0.0
Rapid sequence induction	19	5.8	5	13.9	14	4.8
Modified with mask ventilation	13		3		10	
No mask ventilation	6		2		4	
Cricoid pressure	6	1.8	1	2.8	5	1.7
Use of neuromuscular blockade	190	57.6	27	75.0	163	55.4
Atracurium	113		10		103	
Cis‐atracurium	14		7		7	
Rocuronium	59		10		49	
Succinylcholine	4		1		3	
Vecuronium	3		0		3	
Neuromuscular monitoring						
Yes	26		0		26	
No	164		27		137	
Neuromuscular blockade reversal						
Neostigmine	118		13		105	
Sugammadex	33		4		29	
No reversals	39		10		29	
Maintenance						
Inhalational	262	79.4	30	83.3	232	78.9
Total intravenous anesthesia (TIVA)	51	15.5	6	16.7	45	15.3
Other (e.g., sedation, regional anesthesia only)	17	5.2	0	0.0	17	5.8
Regional anesthesia	69	20.9	7	20.6	62	23.5
With general anesthesia	67		7		60	
With sedation	2		0		2	
Way of provision of regional anesthesia						
Landmarks	49		7		42	
US guided	19		0		19	
Combination of both nerve stimulator and ultrasound	1		0		1	
Type of regional anesthesia						
Caudal	21		7		14	
Craniofacial	4		0		4	
Epidural	6		0		6	
Ilio‐inguinal	4		0		4	
Lower‐limb	10		0		10	
Paravertebral	2		0		2	
Penile	16		0		16	
Spinal	1		0		1	
Transabdominal plane (TAP)	1		0		1	
Rectus sheath (RS)	1		0		1	
Other	2		0		2	
Type of interface for airway management						
Anesthesia (Face) mask	21	6.4	0	0.0	21	7.1
Supraglottic airway (SGA) (e.g., LMA)	94	28.5	4	11.1	90	30.6
Endotracheal tube (ETT)	175	53.0	28	77.8	147	50.0
Non‐invasive positive pressure ventilation (NPPV)	4	1.2	0	0.0	4	1.4
No airway devices (e.g., nasal cannula alone)	33	10.0	3	8.3	30	10.2
Already secured with ETT/SGA/tracheostomy	3	0.9	1	2.8	2	0.7
Use of supraglottic airway (SGA) device						
Inserton at 1st or 2nd attempt	93		4		89	
More than 3rd attempt	1		0		1	
Type of SGA						
Classic	28		2		26	
ProSeal	46		1		45	
Reinforced/ Flexible LMA	6		0		6	
Intubating LMA (ILMA)	1		0		1	
i‐gel	4		1		3	
Other	9		0		9	
Timing of removal of SGA						
Awake	29		0		29	
Semi‐awake	22		4		18	
Deep anesthesia (Deep plane)	43		0		43	
Use of endtracheal tube (ETT)						
Insertion at 1st or 2nd attempt	171		28		143	
More than 3rd attempt	4		0		4	
Cuff of ETT						
Cuffed	108		11		97	
Uncuffed	67		17		50	
Cuff pressure monitoring	57/108		5/11		52/97	
How to intubate						
Direct laryngoscopy	140		22		118	
Video laryngoscopy	31		4		27	
Fiberoptic intubation	1		0		1	
Other	3		2		1	
Intubation way						
Oral	162		28		134	
Nasal	12		0		12	
Through tracheostomy	1		0		1	
Type of ETT						
Normal	130		23		107	
Oral Ring Adair Elwyn (RAE) (“southpolar”)	19		3		16	
Nasal Ring Adair Elwyn (RAE) (“northpolar”)	5		0		5	
Reinforced (“Spiral”)	18		1		17	
Other	3		1		2	
Vocal cords sprayed with lignocaine prior to intubation	8		2		6	
Cormack & Lahane						
Grade I	118		15		103	
Grade II	46		9		37	
Grade III	9		3		6	
Grade IV	1		0		1	
Unknown	1		1		0	
Timing of extubation						
Awake	108		11		97	
Semi‐awake	33		6		27	
Deep‐plane	19		2		17	
Unknown/Not extubated	15		9		6	
Mode of intraoperative ventilation						
Spontaneous ventilation	88		4		84	
Pressure support ventilation	52		3		49	
Continuous mandatory ventilation	190		29		161	
Fluids administration						
Use of glucose containing fluids	56		14		42	
1%	18		7		11	
2.50%	4		1		3	
5%	25		4		21	
10%	4		1		3	
Other%	5		1		4	
Use of colloids	16		7		9	
Synthetic colloids	4		0		4	
Albumin	7		4		3	
Other	5		3		2	
Blood transfusion	16		6		10	
Duration of surgical/ non‐surgical procedure (median, range) [minutes]	60.0	(5.0, 690.0)	61.5	(15.0, 600.0)	60	(5.0, 690.0)
Duration of anesthesia/ sedation (median, range) [minutes]	85.0	(6.0, 805.0)	115	(15.0, 698.0)	82	(6.0, 805.0)
Disposition after anesthesia/sedation						
Ward	166	50.3	11	30.6	155	52.7
Intensive Care Unit	40	12.1	14	38.9	26	8.8
Discharge from PACU (i.e. day‐surgery)	92	27.9	9	25.0	83	28.2
Unknown	32	9.7	2	5.6	30	10.2

*Note*: Patients with severe critical events: *N* = 36 (10.9%). Total number of severe critical events: *N* = 41 (12.4%) (respiratory 23 + cardiovascular 16 + drug error 2).

Among the 330 patients, 36 children (10.9%) experienced at least one SCE, with a total of 41 SCEs occurring during or immediately following anesthesia or sedation. which yields an incidence of 12.4% [95% CI: 9.2–16.4].

Respiratory critical events were observed in 7.0% (23 out of 330) [95% CI: 4.2–9.7] of cases. These included laryngospasm in 3.9% (13 out of 330) [95% CI: 2.3–6.7], bronchospasm in 1.8% (6 out of 330) [95% CI: 0.9–3.8], and pulmonary aspiration in 0.3% (1 out of 330) [95% CI: 0.1–1.7] of cases (Table 4). Post‐anesthetic stridor was observed in 0.9% (3 out of 330) [95% CI: 0.3–1.9] of patients, all of whom were intubated with a cuffed endotracheal tube. The incidence of stridor among these patients was 2.8% (3 out of 108) [95% CI: 0.9–5.7].

**TABLE 4:** Time of occurrence, treatment, and outcome of perioperative respiratory severe critical events (*N* = 23).

	Laryngospasm	Bronchospasm	Pulmonary aspiration	Post‐anesthetic stridor
	(*N* = 13)	(*N* = 6)	(*N* = 1)	(*N* = 3)
Time of occurrence, *n* (%)				
Induction	4 (30.7%)	2 (33.3%)	1 (100%)	‐
Maintenance	3 (23.1%)	2 (33.3%)	‐	‐
Awakening	6 (46.2%)	1 (16.7%)	‐	2 (66.7%)
Post‐anesthesia care unit	‐	1 (16.7%)	‐	1 (33.3%)
Treatment, *n* (%)				
CPAP with face mask	10 (76.9%)	‐	1 (100%)^a^	1 (33.3%)^b^
Propofol	4 (30.8%)	‐	‐	‐
Ventilator with intubation	1 (7.7%)	1 (16.7%)	‐	‐
Bronchodilators	‐	2 (33.3%)	‐	‐
Adrenaline inh	‐	1 (16.7%)	‐	2 (66.7%)
Other	2 (15.4%)	2 (33.3%)	‐	‐
Outcome, *n* (%)				
Transient hypoxemia	8 (61.6%)	3 (50.0%)	‐	‐
Uneventful	5 (38.4%)	3 (50.0%)	‐	2 (66.7%)
Prolonged intubation	‐	‐	1 (100%)	‐
Unplanned ICU admission	‐	‐	‐	1 (33.3%)

Note: Data are *n* (%); there were some repeated events. Airway interventions include application of CPAP, PEEP, or oxygen. Hypoxemia defined as oxygen saturation less than 90% or 10% below the baseline.

Abbreviations: CPAP, continuous positive airway pressure; PEEP, positive end‐expiratory pressure.

^a^ Followed by prolonged intubation.

^b^ Followed by unplanned ICU admission.

Cardiovascular instability requiring intervention was noted in 4.9% [95% CI: 2.9–7.3] of cases. Twelve episodes were attributed to hypotension, three to bleeding, and three to arrhythmias. Treatments included fluid resuscitation (nine episodes), blood transfusion (four episodes), vasopressors (six episodes), and defibrillation in one instance (Table 5). All episodes resolved without significant long‐term sequelae, and no cases of cardiac arrest were reported.

**TABLE 5:** Time of occurrence, type of severe cardiovascular critical events, treatment applied, and outcome (*N* = 16).

Cardiovascular instability	(*N* = 16)
Time of occurrence, *n* (%)	
Induction	7 (43.8%)
Maintenance	8 (50.0%)
Awakening	1 (6.3%)
Post‐anesthesia care unit	1 (6.3%)
Type of event, *n* (%)	
Hypotension	12 (75.0%)
Bleeding	3 (18.8%)
Cardiac arrhythmia	3 (18.8%)
Treatment, *n* (%)	
Fluid resuscitation	9 (56.3%)
Blood products	4 (25.0%)
Vasopressor	6 (37.5%)
Atropine	1 (6.3%)
Defibrillation or electrical cardioversion	1 (6.3%)
Other	1 (6.3%)
Outcome, *n* (%)	
Uneventful	16 (100%)

*Note*: Data are *n* (%); there were some repeated events.

Drug errors were documented in two cases (0.6% [95% CI: 0.1–1.4]), both of which did not result in significant adverse outcomes. Importantly, no instances of anaphylaxis, neurological damage, or cardiac arrest were reported in this pilot study.

### 3.3 Sample size calculation for the main study

Based on the observed SCE rate of 12.4% [95% CI: 9.2–16.4] in the pilot study, the estimated sample size for the main study was calculated as 10 958, using a standard binominal assumption to achieve a 95% confidence level.

## 4 DISCUSSION

### 4.1 Reflection on protocol development

The development of the PEACH in Asia protocol was a collaborative endeavor, significantly strengthened by the support and infrastructure of the ASPA. The ASPA, a robust network with over 700 members across 30 Asian countries, provided a solid foundation for the PEACH study through its long‐standing commitment to advancing pediatric anesthesia care across the region. This extensive network facilitated the integration of diverse perspectives and expertise, ensuring that the protocol was both comprehensive and adaptable to the varied clinical settings across Asia.

Building on the framework established by the successful APRICOT study in Europe,^6^ the PEACH in Asia study protocol was carefully aligned with the established protocol of the APRICOT study to ensure standardized data acquisition and enable comparative analyses. This alignment will enable meaningful comparative discussions and analyses of results across different studies.^12^, ^17^

### 4.2 Informed consent requirements for observational studies

The pilot study highlighted several challenges related to the informed consent process and consent rates in the Philippines. Differences in informed consent requirements for observational studies across countries complicate international research efforts.^18^ In some regions, obtaining informed consent from patients or their guardians is mandatory for all types of studies, including observational ones.^19^ The necessity for written informed consent can introduce selection bias, thereby potentially compromising the validity of the results. To address these challenges in the main study, it is crucial that national and local coordinators implement strategies tailored to low‐literacy populations and ensure that study materials are translated into local languages.^20^ These measures are expected to improve consent rates and enhance the reliability of data collection in the primary research phase.

### 4.3 Assessment of feasibility of data acquisition

The pilot study demonstrated the feasibility of implementing the PEACH in Asia study protocol across nine institutions in nine different countries or regions. The successful enrollment of 330 patients within a three‐day period across most sites indicates that the protocol is robust and can be effectively integrated into varied clinical environments. However, the withdrawal of one institution in Japan due to the burden of data collection underscores the challenges that some sites may face, particularly in resource‐limited settings. This highlights the need for additional support mechanisms, such as enhanced data entry assistance and potentially longer recruitment periods, to ensure successful implementation across all sites.

Data completeness was high, with the majority of patient information accurately recorded and entered into the e‐CRF system. Nevertheless, the pilot study revealed several areas for improvement, such as simplifying the CRF to reduce the workload on clinical staff and addressing technical issues related to e‐CRF entry. The variability in patient enrollment across sites, ranging from 4 to 86 patients, suggests the need for hierarchical logistic and relative risk regression models in the main study to account for institutional diversities and identify independent risk factors for SCEs.^21^, ^22^, ^23^

### 4.4 Outcome measure interpretation of the pilot study

Given the small sample size and inherent limitations of the pilot study, the outcome data should be interpreted with caution. For instance, the observed SCE rate in this pilot study was higher than that reported in the APRICOT study.^6^ This discrepancy may be attributed to selection bias, as the participating hospitals were primarily tertiary care facilities, which typically handle higher‐risk patients. The higher proportion of patients with an ASA physical status classification of III or higher in this pilot likely contributed to the elevated SCE rate. Consequently, the main study must account for this variability in patient severity to ensure accurate and generalizable findings. Increasing the sample size in the main study would enhance the precision of SCE rate estimates and improve the reliability of the results across diverse settings and populations in Asia.

### 4.5 Impact of pilot study results on sample size for main study

The pilot study's findings provide valuable guidance for the design and execution of the main study. The observed rate of SCEs was 12.4% [95% CI: 9.2–16.4], which has been used to estimate the required sample size for the main study. To achieve a 95% confidence level, the sample size has been calculated to need at least 10 958 patients. It is, however, crucial to recognize the inherent uncertainties involved in sample size estimation based on pilot data.^24^, ^25^ The small sample size and the broad confidence intervals suggest that the actual incidence of SCEs may vary. This variability underscores the importance of cautious interpretation of these preliminary estimates. Considering an anticipated 10% non‐response and missing data rate, the adjusted sample size is projected to be 12 184. Given the small pilot sample and wide confidence intervals, these estimates should be interpreted cautiously as the true SCE rate may vary. Additionally, Asia's diversity may necessitate a sample size larger than 10 958 to ensure robust conclusions. Balancing the target sample size and duration of data collection will be key to maintaining the study's validity.

### 4.6 Limitations

This pilot study, conducted across nine institutions in metropolitan areas of nine different countries or regions, has several limitations. The brief recruitment period of just 3 days, combined with variability in patient enrollment across institutions, limits the generalizability of the findings, which is expected given the pilot nature of the study. Additionally, the small sample size constrained our ability to perform statistical comparisons between variables, thus limiting our capacity to draw definitive conclusions.

The substantial socio‐economic, demographic, and cultural diversity among the Asian countries included in the study introduces further challenges.^11^, ^13^, ^14^ Differences in human development indices, anesthesia workforce capacities,^15^ and cultural factors may require specialized analytical approaches for the main study. To address these challenges, the main study will employ hierarchical logistic regression and relative risk models to identify independent risk factors for SCEs, while accounting for variations at national, regional, and institutional levels.^21^, ^22^, ^23^

## 5 CONCLUSIONS

The PEACH in Asia study project has been successfully initiated to explore severe critical events (SCEs) in pediatric anesthesia across Asia. Leveraging the extensive network of the Asian Society of Pediatric Anesthesiologists (ASPA), the protocol was carefully crafted to be feasible across diverse practices while aligning with existing cohort studies such as the APRICOT study. The pilot study confirmed the viability of a standardized approach, highlighting the advantages of a region‐wide data collection strategy. However, challenges, particularly in resource‐limited settings, were identified and must be addressed for the success of the main study. The pilot study also provided preliminary data for estimating the required sample size for the main study. By refining the study design and overcoming the challenges, the PEACH in Asia study aspires to yield robust data that will substantially advance the understanding of pediatric anesthesia safety in Asia, ultimately facilitating the development of targeted interventions and best practices tailored to the unique healthcare needs in Asia.

## FUNDING INFORMATION

This research work, as part of the PEACH in Asia study project, was supported by Kawano Masanori Memorial Public Interest Incorporated Foundation for Promotion of Pediatrics in Japan (2022–2023) and has been supported by MEXT KAKENHI Grant Number 24 K13535 (since 2024).

## CONFLICT OF INTEREST STATEMENT

All the authors are members of the Asian Society of Pediatric Anaesthesiologists (ASPA) research special interest group. SZU Ozgen is the current president of the ASPA and the current chair of pediatric anesthesia committee for the World Federations of Societies of Anaesthesiologists WFSA. Otherwise, the authors declare that they have no conflicts of interest to disclose.

## ETHICS STATEMENT

This study was conducted in compliance with all ethical principles of the Declaration of Helsinki and International Conference on Harmonization Good Clinical Practice guidelines. The Institutional Review Board at Tokyo Metropolitan Ohtsuka Hospital, Tokyo, Japan gave ethical approval for the PEACH in Asia study project as an international prospective observational multicenter study. Ethics requirement differed among countries and even within a given country. All participating institutions received formal ethics approval or a waiver, as appropriate.

## CLINICAL TRIAL REGISTRATION

PEACH in Asia study is registered as an international prospective observational multicenter observational study in the University hospital Medical Information Network (UMIN) (UMIN Clinical Trials Registry identifier # UMIN000046328: registered on December 28th, 2021).

## Supporting information


Appendix S1.

## Data Availability

The data that support the findings of this study are available from the corresponding author upon reasonable request.
