# Supplementary material for: Protocol development and feasibility of the PEACH in Asia study: A pilot study on PEri‐anesthetic morbidity in CHildren in Asia
Source: Paediatr Anaesth. 2024 Nov 9;35(2):125–39. doi: 10.1111/pan.15034 (PMC11701951; doi:10.1111/pan.15034)
Supplement: Supplementary file 1 — Appendix S1. [file PAN-35-125-s001.pdf]

CRF 1: Preoperative Data (Before Anesthesia)

PATIENT INFORMATION & CONSENT		
1.1	Country or Region Name	
	(choose single most appropriate)<br>☐ 001: Afghanistan<br>☐ 002: Bahrain<br>☐ 003: Bangladesh<br>☐ 004: Bhutan<br>☐ 005: Brunei Darussalam<br>☐ 006: Cambodia<br>☐ 007: China<br>☐ 008: Democratic People's Republic of Korea<br>☐ 009: India<br>☐ 010: Indonesia<br>☐ 011: Iran (Islamic Republic of)<br>☐ 012: Iraq<br>☐ 013: Japan<br>☐ 014: Jordan<br>☐ 015: Kuwait<br>☐ 016: Lao People's Democratic Republic<br>☐ 017: Lebanon<br>☐ 018: Malaysia<br>☐ 019: Maldives	☐ 020: Mongolia<br>☐ 021: Myanmar<br>☐ 022: Nepal<br>☐ 023: Oman<br>☐ 024: Pakistan<br>☐ 025: Philippines<br>☐ 026: Qatar<br>☐ 027: Republic of Korea<br>☐ 028: Saudi Arabia<br>☐ 029: Singapore<br>☐ 030: Sri Lanka<br>☐ 031: Syrian Arab Republic<br>☐ 032: Thailand<br>☐ 033: Timor-Leste<br>☐ 034: Turkey<br>☐ 035: United Arab Emirates<br>☐ 036: Viet Nam<br>☐ 037: Yemen<br>☐ 038: Hong Kong<br>☐ 039: Chinese Taipei
1.2	Hospital code	|__|__|__| (3 digit)<br>(Provided by the sponsor of this study)
1.3	Individual patient number at your hospital	|__|__|__| (3 digit)<br>(You can give a number in the order you collect the data in your hospital)
2.1	Informed consent applicable?<br>(choose “No” if waived by local ethics committee or local research board)	☐ Yes<br>☐ No
2.2	If yes, was consent obtained?	☐ Yes ☐ No ☐ N/A
2.3	If obtained, enter date of Informed Consent	|__|__|__|__ |-|__|__|- |__|__| [YYYY – MM – DD]<br><br>☐ N/A

✂---

Study Subject ID: |__|__|__|-|__|__|__|-|__|__|__|

DEMOGRAPHICS		
3.1	Date of Anesthesia / Sedation	|__|__|__|__ |-|__|__|- |__|__| [YYYY – MM – DD]
	3.1.1 Anesthesia / Sedation time	*(choose single most appropriate)*<br>☐ Opening hours of Operating room (i.e. Daytime of Weekdays)<br>☐ After hours/ Weekends/ Holidays
	3.1.2 Degree of Urgency	*(choose single most appropriate)*<br>☐ Elective ☐ Emergency
3.2	Was the child premature?<br>(less than 37 weeks)	☐ Yes<br>☐ No
	3.2.1<br>If yes, gestational age at birth?	|__|__| weeks [21-36]<br><br>☐ N/A
3.3	Age of the patient	*(choose single most appropriate)*<br>☐ **Preterm neonatal:** The period at birth when a newborn is born before the full gestational period<br>☐ **from birth ≦27 days** (Term neonatal)<br>☐ **≧28 days, ≦12 months** (Infancy)<br>☐ **≧13 months, < 2 years** (Toddler)<br>☐ **≧2 years, ≦5 years** (Early childhood)<br>☐ **≧6 years, ≦11 years** (Middle childhood)<br>☐ **≧12 years, ≦15 years** (Early adolescence)
3.4	Sex of the patient	☐ Male ☐ Female ☐ Unknown
3.5	Ethnicity of the patient	*(choose single most appropriate)*<br>☐ White<br>☐ Spanish/Hispanic/Latino<br>☐ Asian (e.g. Indian, Pakistani, Bangladeshi, Chinese, Vietnam, etc.)<br>☐ Black (Caribbean, African)<br>☐ Arabic (North Africa, Middle East)<br>☐ Other
3.6	ASA Physical Status	☐ 1 ☐ 2 ☐ 3 ☐ 4 ☐ 5<br>☐ 1E ☐ 2E ☐ 3E ☐ 4E ☐ 5E
3.7	Height of the patient	|__|__|__| cm [30-200]<br><br>☐ Not available
3.8	Weight of the patient	|__|__|__| . |__| kg [0.0-150.0]<br>**Please write numbers with 1 decimal places.**<br>(e.g. If the body weight of your patient is "**15**" kg, you need to write down "**15.0**" kg.)<br><br>☐ Not available

MEDICAL HISTORY		
4.1	Flu/cold:<br>Does child currently (or in the 2 weeks preceding procedure) have flu or a cold?	☐ Yes ☐ No ☐ Not available
4.2	Wheezing/whistling:<br>Has the child had wheezing or whistling spontaneously or after exercise in the last 12 months?	☐ Yes ☐ No ☐ Not available
4.3	Asthma:<br>Has the child ever had diagnosed asthma?	☐ Yes ☐ No ☐ Not available
4.4	Atopy:<br>Is child atopic? (Sneezing, or a runny nose or itchy watery eyes or nose or itchy rash or eczema, in the last 12 months)?	☐ Yes ☐ No ☐ Not available
4.5	Allergy:<br>Has the child ever had allergy?	☐ Yes ☐ No ☐ Not available
	4.5.1<br>If yes, indicate all allergies that apply	☐ Food ☐ Nut ☐ Latex<br>☐ Antibiotics ☐ Other<br><br>☐ Not applicable (e.g. The patient has NO allergies)
4.6	Snoring:<br>While sleeping, does the child snore?	☐ Yes ☐ No ☐ Not available
4.7	Smoking:<br>Does anyone in the family/ giving care to the child smoke	☐ Yes ☐ No ☐ Not available
4.8	Anesthetic complication:<br>Has the child had any previous documented anesthetic complication?	☐ Yes ☐ No ☐ Not available
4.9	Medication:<br>Does the child take any regular medication, natural products and/or homeopathic products?	☐ Yes ☐ No ☐ Not available
4.10	Handicap:<br>Does the child have metabolic/ genetic disorder or neurological impairment?	☐ Yes ☐ No ☐ Not available

INDICATION		
5.1	Type of procedure	☐ Surgical<br>☐ Non- Surgical procedure
	5.1.1<br>If surgical,<br>indicate type of surgical procedure	*(tick all that apply)*<br>☐ Neurosurgery<br>☐ Head and Neck<br>☐ Ophthalmology<br>☐ Ear-Nose-Throat<br>☐ Plastics (including cleft palate & lip)<br>☐ Cutaneous/Dermatology<br>☐ Cardiac surgery<br>☐ Thoracic<br>☐ Gastro/Abdominal<br>☐ Hepato-biliary/Pancreas<br>☐ Urological/Kidney<br>☐ Orthopedic<br>☐ Trauma<br><br>☐ Not applicable (i.e. Non-Surgical procedure)
	5.1.2<br>If non-surgical,<br>indicate type of non-surgical procedure	*(tick all that apply)*<br>☐ Ophthalmologic examination<br>☐ Dental<br>☐ Bronchoscopy<br>☐ Gastroenterology<br>☐ Biopsy<br>☐ Bone Marrow & Lumbar puncture<br>☐ CT-Scan<br>☐ MRI (Magnetic rad. Imaging)<br>☐ Venous access<br>☐ Burns dressing<br>☐ Other non-surgical<br><br>☐ Not applicable (i.e. Surgical procedure)
5.3	Patient type	*(choose single most appropriate)*<br>☐ Outpatient ☐ Inpatient

ANAESTHESIA PLAN		
6.1	The senior anesthesiologist in charge<br>What kind of Anesthesiologist?<br><br>*"the senior anesthesiologist in charge" means "the person who directly and mainly handles anesthesia or sedation of the patient as in-charge, NOT the person who supervised it."*	*(choose single most appropriate)*<br>☐ Specialist anesthesiologist with mainly pediatric practice (>50%)<br>☐ Specialist anesthesiologist with occasional pediatric anesthesia cases (<50%)<br>☐ Anesthesiologist in training<br>☐ Anesthetic nurse
	6.1.1 Experience<br>For how many years the senior person in charge of the patient has been practicing?<br><br>*the definition of "years of experience" here is "no. of years since starting anesthesia training (including the period of anesthesia training)"*	|__|__| yrs [0-60]
6.2	Pre-Medications<br>Was there any medication taken by the child just before the anesthesia?	☐ Yes<br>☐ No
	6.2.1<br>If yes, indicate medication(s):	*(tick all that apply)*<br>☐ Midazolam oral<br>☐ Midazolam nasal<br>☐ Midazolam rectal<br>☐ Clonidine oral<br>☐ Clonidine nasal<br>☐ Dexmedetomidine oral<br>☐ Dexmedetomidine nasal<br>☐ Acetaminophen (Paracetamol) oral<br>☐ Acetaminophen (Paracetamol) rectal<br>☐ Local anesthetic cream/ tape (e.g. EMLA)<br>☐ Other<br><br>☐ Not applicable (i.e. no pre-medications)
6.3	Parental presence<br>Was the child accompanied by<br>a parent or guardian during the induction?	☐ Yes<br>☐ No
6.4	Monitoring<br>Please specify	*(tick all that apply)*<br>☐ SpO_2 ☐ ECG ☐ NIBP<br>☐ Capnography ☐ end-tidal anesthetic gas<br>☐ Invasive arterial blood pressure (ABP)<br>☐ Central venous pressure (CVP)<br>☐ Electroencephalogram derivated data (e.g. BIS)<br>☐ Near infrared spectroscopy (NIRS)<br>☐ Neuromuscular monitoring<br>☐ Others

CRF 2: Intraoperative Data (During anesthesia and 60 minutes afterwards)

INDUCTION		
7.1	Induction type at onset	*(Choose single most appropriate)*<br>☐ Inhalational<br>☐ Intravenous<br>☐ Intramuscular
7.2	Rapid Sequence Induction	*(Choose single most appropriate)*<br>☐ No mask ventilation<br>☐ Modified with mask ventilation<br><br>☐ Not applicable (i.e. Not rapid sequence induction)
7.3	Cricoid pressure	☐ Yes<br>☐ No
7.4	Induction medication	*(tick all that apply)*<br>☐ Sevoflurane<br>☐ Halothane<br>☐ Nitrous Oxide<br>☐ Propofol<br>☐ Barbiturates (e.g. Thiopentone)<br>☐ Etomidate<br>☐ Ketamine<br>☐ Dexmedetomidine<br>☐ Opioid(s)<br>☐ Other
7.5	Use of neuromuscular blocking agent(s) (NMBs) at induction and/ or during maintenance	*(tick all that apply)*<br>☐ None<br>☐ Succinylcholine<br>☐ Rocuronium<br>☐ Vecuronium<br>☐ Cis-atracurium<br>☐ Atracurium<br>☐ Other
	7.5.1<br>If NMBs used,<br>neuromuscular monitoring?	☐ Yes<br>☐ No<br><br>☐ Not applicable (i.e. No NMBs were used)
	7.5.2<br>If NMBs used, reversal at the end?	*(choose single most appropriate)*<br>☐ Neostigmine<br>☐ Sugammadex<br>☐ No reversal<br><br>☐ Not applicable (i.e. No NMBs were used)

MAINTENANCE		
8.1	Anesthesia type during maintenance	*(choose single most appropriate)*<br>☐ Inhalational<br>☐ Total intravenous anesthesia (TIVA)<br>☐ Other (e.g. Sedation, Regional anesthesia alone)
8.2	Medication during maintenance	*(tick all that apply)*<br>☐ Sevoflurane<br>☐ Halothane<br>☐ Isoflurane<br>☐ Desflurane<br>☐ Nitrous Oxide<br>☐ Propofol<br>☐ Barbiturates (e.g. Thiopentone)<br>☐ Ketamine<br>☐ Dexmedetomidine<br>☐ Opioid(s)<br>☐ Other
8.3	Regional anesthesia<br>by anesthesiologist	*(Choose single most appropriate)*<br>▢ Alone<br>▢ With sedation<br>▢ With general anesthesia<br>▢ None
	8.3.1<br>If regional anesthesia done,<br>specify type	*(Choose single most appropriate)*<br>☐ Nerve stimulation (NS)<br>☐ Ultrasound (US) guided<br>☐ Combination of both NS and US<br>☐ Landmarks<br><br>☐ Not applicable (i.e. No regional anesthesia done)
	8.3.2<br>If regional anesthesia done,<br>specify type of neuraxial block:	*(tick all that apply)*<br>☐ Spinal<br>☐ Epidural (except for Caudal)<br>☐ Caudal<br>☐ Upper limb<br>☐ Lower limb<br>☐ Paravertebral<br>☐ Intercostal<br>☐ Ilioinguinal<br>☐ TAP (i.e. Transabdominal plane)<br>☐ Rectus sheath (periumbilical)<br>☐ Penile<br>☐ Pudendal<br>☐ Craniofacial<br>☐ Other<br><br>☐ Not applicable (i.e. No regional anesthesia done)

AIRWAY MANAGEMENT		
9.1	Specify type of interface for airway management	*(Choose single most appropriate)*<br>☐ Anesthesia (Face) mask<br>☐ Supraglottic airway (SGA) (e.g. LMA)<br>☐ Endotracheal tube (ETT)<br>☐ Non-invasive positive pressure ventilation (NPPV)<br>(e.g. nasal CPAP, high-flow nasal cannula)<br>☐ No airway devices (e.g. nasal cannula alone)<br>☐ Already secured with ETT/ SGA/ tracheostomy

9.2	Use SGA?	☐ Yes ☐ No
	9.2.1<br>If SGA used, insertion at	*(Choose single most appropriate)*<br>☐ 1st or 2nd attempt<br>☐ more than 3rd attempt<br><br>☐ Not applicable (i.e. No SGA used)
	9.2.2<br>If SGA used, type of SGA	*(Choose single most appropriate)*<br>☐ Classic<br>☐ ProSeal<br>☐ Reinforced/ Flexible LMA<br>☐ Intubating LMA (ILMA)<br>☐ iGel<br>☐ Other<br><br>☐ Not applicable (i.e. No SGA used)
	9.2.3<br>Removal of SGA	*(Choose single most appropriate)*<br>☐ Awake<br>☐ Semi-awake<br>☐ Deep anesthesia (Deep plane)<br><br>☐ Not applicable (i.e. No SGA used)

9.3	Use ETT?	☐ Yes ☐ No
	9.3.1<br>If ETT used, insertion at	*(Choose single most appropriate)*<br>☐ 1st or 2nd attempt<br>☐ more than 3rd attempt<br><br>☐ Not applicable (i.e. No ETT used)
	9.3.2<br>If ETT used, type of ETT	*(Choose single most appropriate)*<br>☐ Cuffed<br>☐ Uncuffed<br><br>☐ Not applicable (i.e. No ETT used)
	9.3.3<br>If cuffed ETT used, monitoring cuff pressure?	*(Choose single most appropriate)*<br>☐ Yes<br>☐ No<br><br>☐ Not applicable (i.e. No cuffed ETT used)

	9.3.4<br>Intubation	*(Choose single most appropriate)*<br>☐ Direct laryngoscopy<br>☐ Video laryngoscopy<br>☐ Through SGA (e.g. ILMA)<br>☐ Fiberoptic intubation<br>☐ Other<br><br>☐ Not applicable (i.e. No ETT used)
	9.3.5<br>Intubation way	*(Choose single most appropriate)*<br>☐ Oral<br>☐ Nasal<br>☐ Through tracheostomy<br><br>☐ Not applicable (i.e. No ETT used)
	9.3.6<br>Tube type	*(Choose single most appropriate)*<br>☐ Classic (Normal)<br>☐ Oral Ring–Adair–Elwyn (RAE) (“southpolar”)<br>☐ Nasal Ring–Adair–Elwyn (RAE) (“northpolar”)<br>☐ Reinforced (“Spiral”)<br>☐ Other<br><br>☐ Not applicable (i.e. No ETT used)
	9.3.7<br>Vocal Cords sprayed with lignocaine (lidocaine) prior to intubation?	☐ Yes<br>☐ No<br><br>☐ Not applicable (i.e. No ETT used)
	9.3.8<br>Cormack-Lehane score	*(Choose single most appropriate)*<br>☐ 1 ☐ 2 ☐ 3 ☐ 4
	9.3.9<br>Removal of ETT	*(Choose single most appropriate)*<br>☐ Awake<br>☐ Semi-awake<br>☐ Deep anesthesia (Deep plane)<br><br>☐ Not applicable (i.e. No ETT used)

VENTILATION		
10.	Ventilation type	*(Choose single most appropriate)*<br>☐ Spontaneous ventilation<br>☐ Pressure support ventilation (PSV)<br>☐ Continuous mandatory ventilation (CMV)
10.1	If CMV used, specify the type	*(Choose single most appropriate)*<br>☐ Pressure controlled<br>☐ Volume controlled<br>☐ Pressure regulated volume controlled<br>☐ Other<br><br>☐ Not applicable (i.e. No CMV used)

FLUIDS		
11	Fluids administered intravenously during anesthesia / sedation?	☐ Yes ☐ No
11.1	Glucose-containing fluids administered?	☐ Yes ☐ No<br>☐ Not applicable (i.e. No IV fluids used)
	11.1.1<br>If Glucose-containing fluids used, provide concentration	*(Choose single most appropriate)*<br>☐ 1%<br>☐ 2.5%<br>☐ 5%<br>☐ 10%<br>☐ Other %<br>☐ Not applicable (i.e. No IV fluids used or No Glucose-containing fluids used)
11.2	IV Colloids administered?	*(Choose single most appropriate)*<br>☐ No<br>☐ Albumin<br>☐ Synthetic colloids<br>☐ Other<br>☐ Not applicable (i.e. No IV fluids used)
11.3	Blood product administered?	*(Choose single most appropriate)*<br>☐ Yes ☐ No<br>☐ Not applicable (i.e. No IV fluids used)
	11.3.1<br>If blood products used, type of blood products	*(tick all that apply)*<br>☐ Packed red blood cells (pRBCs)<br>☐ Fresh frozen plasma (FFP)<br>☐ Platelets<br>☐ Fibrinogen<br>☐ Cryoprecipitate<br>☐ Other<br>☐ Not applicable (i.e. No blood products used)

TIME		
12.1	Duration of surgical/ non-surgical procedure	|__|__|__|__| min [1-9999]
12.2	Duration of anesthesia/ sedation	|__|__|__|__| min [1-9999]

CRF 3: Perioperative Complications during anesthesia and up to 60 minutes afterwards
For definitions of Complications please refer to last page of CRF or appendix of protocol

13.
Any perioperative complications during anesthesia and up to 60 minutes afterwards?
☐ Yes
☐ No
(As for the definitions of perioperative complications, please refer to the end of the case report form)

13.1	Bronchospasm?	☐ Yes ☐ No
13.1.1	Bronchospasm, Time of occurrence?<br>*(tick all that apply)*	☐ Induction<br>☐ Maintenance<br>☐ Awakening<br>☐ Post-anesthesia care unit (PACU) or Ward<br><br>☐ Not applicable (i.e. No Bronchospasm)
13.1.2	Bronchospasm, specify Treatment:<br>*(tick all that apply)*	☐ Ventilation with intubation<br>☐ Bronchodilator<br>☐ Adrenaline (Epinephrine)<br>☐ Other<br><br>☐ Not applicable (i.e. No Bronchospasm)
13.1.3	Bronchospasm, Outcome of event?<br>*(tick all that apply)*	☐ Uneventful<br>☐ Hypoxemia (SpO_2 < 90% or 10% below the baseline)<br>☐ Prolonged intubation<br>☐ Cardiac arrest<br><br>☐ Not applicable (i.e. No Bronchospasm)

13.2	Laryngospasm?	☐ Yes ☐ No
13.2.1	Laryngospasm, Time of occurrence?<br>*(tick all that apply)*	☐ Induction<br>☐ Maintenance<br>☐ Awakening<br>☐ Post-anesthesia care unit (PACU) or Ward<br><br>☐ Not applicable (i.e. No Laryngospasm)
13.2.2	Laryngospasm, specify Treatment:<br>*(tick all that apply)*	☐ Ventilation with intubation<br>☐ CPAP with anesthesia (face) mask<br>☐ Propofol<br>☐ Opioid(s)<br>☐ Neuromuscular blockade(s)<br>☐ Other<br><br>☐ Not applicable (i.e. No Laryngospasm)
13.2.3	Laryngospasm, Outcome of event?<br>*(tick all that apply)*	☐ Uneventful<br>☐ Hypoxemia (SpO2 < 90% or 10% below the baseline)<br>☐ Prolonged intubation<br>☐ Pulmonary edema<br>☐ Cardiac arrest<br><br>☐ Not applicable (i.e. No Bronchospasm)

✂---
Study Subject ID: |__|__|__|-|__|__|__|-|__|__|__|

13.3	Pulmonary aspiration?	☐ Yes ☐ No
13.3.1	Pulmonary aspiration, Time of occurrence?<br>*(tick all that apply)*	☐ Induction<br>☐ Maintenance<br>☐ Awakening<br>☐ Post-anesthesia care unit (PACU) or Ward<br><br>☐ Not applicable (i.e. No Pulmonary aspiration)
13.3.2	Pulmonary aspiration, specify Treatment:<br>*(tick all that apply)*	☐ Broncho-tracheal suction<br>☐ Intubation<br>☐ CPAP<br>☐ Bronchodilator<br>☐ Other<br><br>☐ Not applicable (i.e. No Pulmonary aspiration)
13.3.3	Pulmonary aspiration, Outcome of event?<br>*(tick all that apply)*	☐ Uneventful<br>☐ Hypoxemia (SpO2 < 90% or 10% below the baseline)<br>☐ Prolonged intubation<br>☐ Cardiac arrest<br><br>☐ Not applicable (i.e. No Pulmonary aspiration)

13.4	Drug error?	☐ Yes ☐ No
13.4.1	Drug error, Time of occurrence?<br>*(tick all that apply)*	☐ Induction<br>☐ Maintenance<br>☐ Awakening<br>☐ Post-anesthesia care unit (PACU) or Ward<br><br>☐ Not applicable (i.e. No Drug errors)
13.4.2	Drug error, specify Type:<br>*(tick all that apply)*	☐ Wrong dosage<br>☐ Wrong product<br>☐ Wrong site of administration<br>☐ Other<br><br>☐ Not applicable (i.e. No Drug errors)
13.4.3	Drug error, Treatment necessary?	☐ Yes ☐ No<br>☐ Not applicable (i.e. No Drug errors)
13.4.3	Drug error, Outcome of event?<br>*(tick all that apply)*	☐ Uneventful<br>☐ Unplanned admission to ICU or Ward<br>☐ Cardiac arrest<br>☐ Other<br><br>☐ Not applicable (i.e. No Drug errors)

13.5	Anaphylaxis?	☐ Yes ☐ No
13.5.1	Anaphylaxis, Time of occurrence?<br>*(tick all that apply)*	☐ Induction<br>☐ Maintenance<br>☐ Awakening<br>☐ Post-anesthesia care unit (PACU) or Ward<br><br>☐ Not applicable (i.e. No Anaphylaxis)
13.5.2	Anaphylaxis, specify Treatment:<br>*(tick all that apply)*	☐ Fluid resuscitation<br>☐ Intramuscular Adrenaline (Epinephrine)<br>☐ Intravenous Adrenaline (Epinephrine)<br>☐ Bronchodilator<br>☐ Intubation, followed by mechanical ventilation<br>☐ Cardiopulmonary resuscitation<br><br>☐ Not applicable (i.e. No Anaphylaxis)
13.5.3	Anaphylaxis, Outcome of event?<br>*(tick all that apply)*	☐ Uneventful<br>☐ Pulmonary edema<br>☐ Cardiac arrest<br>☐ Prolonged intubation<br><br>☐ Not applicable (i.e. No Anaphylaxis)

13.6	Cardiovascular instability?	☐ Yes ☐ No
13.6.1	Cardiovascular instability, Time of occurrence?<br>*(tick all that apply)*	☐ Induction<br>☐ Maintenance<br>☐ Awakening<br>☐ Post-anesthesia care unit (PACU) or Ward<br><br>☐ Not applicable (i.e. No Cardiovascular instability)
13.6.2	Cardiovascular instability, specify Type:<br>*(tick all that apply)*	☐ Cardiac arrhythmia<br>☐ Hypotension<br>☐ Bleeding<br>☐ Other (e.g. Vasodilation)<br><br>☐ Not applicable (i.e. No Cardiovascular instability)
13.6.3	Cardiovascular instability, specify Treatment:<br>*(tick all that apply)*	☐ Fluid resuscitation<br>☐ Blood product<br>☐ Vasopressor<br>☐ Atropine<br>☐ Defibrillation or electrical cardioversion<br>☐ Other<br><br>☐ Not applicable (i.e. No Cardiovascular instability)
13.6.4	Cardiovascular instability, Outcome of event?<br>*(tick all that apply)*	☐ Uneventful<br>☐ Coagulopathy<br>☐ Cardiac arrest<br>☐ Unplanned admission to ICU or Ward<br><br>☐ Not applicable (i.e. No Cardiovascular instability)

13.7	Neurological damage(s)?	☐ Yes ☐ No
13.7.1	Neurological damage, Time of occurrence?<br>*(tick all that apply)*	☐ Induction<br>☐ Maintenance<br>☐ Awakening<br>☐ Post-anesthesia care unit (PACU) or Ward<br><br>☐ Not applicable (i.e. No Neurological damage)
13.7.2	Neurological damage, Treatment necessary?	☐ Yes ☐ No<br>☐ Not applicable (i.e. No Neurological damage)
13.7.3	Neurological damage, Outcome of event?<br>*(tick all that apply)*	☐ Uneventful<br>☐ Unplanned admission to ICU or Ward<br>☐ Poor neurological outcome<br>☐ Death<br><br>☐ Not applicable (i.e. No Neurological damage)

13.8	Cardiac arrest?	☐ Yes ☐ No
13.8.1	Cardiac arrest, Time of occurrence?<br>*(tick all that apply)*	☐ Induction<br>☐ Maintenance<br>☐ Awakening<br>☐ Post-anesthesia care unit (PACU) or Ward<br><br>☐ Not applicable (i.e. No Cardiac arrest)
13.8.2	Cardiac arrest, specify Treatment:<br>*(tick all that apply)*	☐ Closed chest compression<br>☐ Open (Direct) cardiac massage<br>☐ Defibrillation<br>☐ Adrenaline (Epinephrine)<br>☐ Extracorporeal membrane oxygenation (i.e. E-CPR)<br><br>☐ Not applicable (i.e. No Cardiac arrest)
13.8.3	Cardiac arrest, Outcome of event?<br>*(tick all that apply)*	☐ Uneventful<br>☐ Unplanned admission to ICU or Ward<br>☐ Poor neurological outcome<br>☐ Death<br><br>☐ Not applicable (i.e. No Cardiac arrest)

13.9	Postoperative stridor?	☐ Yes ☐ No
13.9.1	Stridor, Time of occurrence?<br>*(tick all that apply)*	☐ Awakening<br>☐ Post-anesthesia care unit (PACU) or Ward<br><br>☐ Not applicable (i.e. No Stridor)
13.9.2	Stridor, specify Treatment:<br>*(tick all that apply)*	☐ CPAP<br>☐ Adrenaline (Epinephrine)<br>☐ Other<br><br>☐ Not applicable (i.e. No Stridor)
13.9.3	Stridor, Outcome of event?<br>*(tick all that apply)*	☐ Uneventful<br>☐ Unplanned admission to ICU or Ward<br>☐ Intubation<br>☐ Not applicable (i.e. No Stridor)

CRF 4: Postoperative Data
Disposition after anesthesia/ sedation care

14.1	Where was the patient transferred after the anesthesia/ sedation care?	*(Choose single most appropriate)*<br>☐ Discharge from PACU (i.e. day-surgery)<br>☐ Ward<br>☐ Intensive care unit (ICU)
14.2	Oxygen delivery?<br><br>*Some institutions or anesthesiologists give oxygen after general anesthesia "routinely" or "as a routine practice." At that time, you can select the answer "Yes, systematic" (= routinely), which should be different from "Yes, if necessary" (= as needed).*	*(Choose single most appropriate)*<br>☐ Yes, systematic<br>☐ Yes, if necessary<br>☐ No

Appendix – Critical Events Definitions
(PEACH in Asia study)

1	**Bronchospasm**	An increased respiratory effort, especially during expiration, and wheeze on auscultation.<br>If the patient is ventilated, bronchospasm may also be considered if a significant increase in peak inspiratory pressure (under volume controlled ventilation) or significant decrease in tidal volume (under pressure controlled ventilation) are observed. In all cases, any episode of airway constriction requiring the administration of a bronchodilator will be recorded.
2	**Laryngospasm**	Complete airway obstruction associated with rigidity of the abdominal and chest walls and leading to unsuccessful child's ventilation, or glottic closure associated with chest movement but silent unsuccessful child's respiratory efforts and assisted ventilation, unrelieved in both situations with simple jaw thrust and continuous positive airway pressure(CPAP) maneuvers and requiring the administration of medication (propofol, fentanyl, suxamethonium, rocuronium etc.) and/ or tracheal intubation.
3	**Pulmonary aspiration**	The presence of any non-respiratory secretions (bilious or particulate) in the airway as evidenced by laryngoscopy, suctioning, or bronchoscopy.<br>In a situation where there was suspicion of pulmonary aspiration but no positive aspiration of non-respiratory secretions, new clinical and/ or chest X-ray signs consistent with aspiration are accepted as evidence for it (e.g., new wheeze or crackles in the chest after regurgitation or vomiting incident).
4	**Drug error**	The administration of a wrong drug, or a wrong dose given by any route, or a wrong site of administration, that has led to either respiratory/ cardiac/ neurological consequence or to an unplanned admission to the intensive care unit(ICU) or prolonged hospitalization.
5	**Anaphylaxis**	The occurrence of any suspected IgE or non-IgE mediated severe allergic reaction leading to cardiovascular instability and/or severe bronchospasm and requiring immediate resuscitation (fluid resuscitation and epinephrine).
6	**Cardiovascular instability**	The occurrence of either one of the following:<br>1. **cardiac arrhythmia** defined as electrocardiogram(ECG) evidence of cardiac rhythm disturbance considered by clinical staff to be severe enough to require treatment (e.g. anti-arrhythmic agents, vasoactive agents, intravenous fluid, etc.). This includes arrhythmias occurring following regional analgesia and requiring intervention. For example: bradycardia requiring atropine, supraventricular tachycardia, atrial or ventricular tachyarrhythmia, torsade de pointe, etc.<br>2. **hypotension** defined as a drop in blood pressure requiring intervention by the anesthesiologist (fluid resuscitation and/or the administration of vasoactive drugs).<br>3. **bleeding** resulting in hypotension and necessitating unanticipated and unpredicted blood transfusion.<br>4. **cardiovascular instability** despite anticipated bleeding and transfusion (e.g.: liver transplant, scoliosis…).
7	**Neurological damage**	1. In case of regional anaesthesia: the occurrence of nerve injury or spinal cord insult or seizure requiring resuscitation.<br>2. In case of general anaesthesia: any episode of seizure, pressures sore, episodes of loss of vision or new onset of central neurological impairment.<br>This includes peripheral nerve injury following positioning (ulnar nerve, external popliteal nerve) or puncture (median or ulnar nerve).

✂--
Study Subject ID: |__|__|__|-|__|__|__|-|__|__|__|

8	**Cardiac Arrest (Peri-anesthetic)**	Cessation of circulation (e.g. pulseless electric activity, asystole, ventricular fibrillation/tachycardia) requiring open or closed chest compressions, or resulting in death, while the patient is in the care of the anesthetic team.
9	**Stridor (Post-operative)**	A severe inspiratory flow limitation with sternal retraction, intrathoracic pressure swing, and potentially cyanosis occurring in the post-anesthesia care unit(PACU) and necessitating the administration of oxygen, intravenous steroids and/or epinephrine (nebulization) or tracheal intubation.
